# PROVIDING STUDENTS WITH INSIGHTS ABOUT AGING AND TECHNOLOGY USING A COLLABORATIVE TEACHING APPROACH

**DOI:** 10.1093/geroni/igae098.1391

**Published:** 2024-12-31

**Authors:** Elinor Schoenfeld, Patricia Bruckenthal, Shelley Horwitz, Erez Zadok, Tracy Trimboli, Fan Ye

**Affiliations:** Stony Brook University School of Medicine, Stony Brook, New York, United States; Stony Brook University, Stony Brook, New York, United States; Stony Brook University, Stony Brook, New York, United States; Stony Brook University, Stony Brook, New York, United States; Stony Brook University, Stony Brook, New York, United States; Stony Brook University, Stony Brook, New York, United States

## Abstract

Novel and innovative methods are needed to introduce our youth to what roles they can play in supporting our aging population, whether it be in a professional or personal role. To engage college lowerclassmen in discussions about aging, a multidisciplinary team of faculty from the College of Engineering and Applied Sciences and the Schools of Medicine, Nursing, and Social Welfare developed a 10-week seminar entitled “Smart Aging – at the Intersection of Technology and Healthcare.” Students were introduced to community engagement, modern sensing, and analytics technologies for physiologic and physical data, concerns about data security and privacy issues related to technology use, and issues faced by older adults aging at home, including social isolation and social determinants of health. Students learned the process of developing vignettes/personas related to aging in place and how technology may help overcome an identified aging-in-place challenge. To learn about aging-in-place outside of class, students were asked to speak with family members or neighbors about aging. Each week students were asked to share and discuss what they have learned about aging in place from their explorations. At the end of the course, students presented their own vignettes/personas and graded the other students’ work. We are currently taking lessons learned to develop a similar multidisciplinary graduate-level course. During this presentation, we will share our experiences with teaching a cohort of freshman and sophomores about aging and share some of their vignettes/personas to highlight their perceptions about and insights into aging.

